# Supplementary material for: Substrate binding and catalytic mechanism of the Se-glycosyltransferase SenB in the biosynthesis of selenoneine
Source: Nat Commun. 2024 Feb 23;15:1659. doi: 10.1038/s41467-024-46065-6 (PMC10891094; doi:10.1038/s41467-024-46065-6)

Supplementary Information for

Substrate binding and catalytic mechanism of the *Se*-glycosyltransferase SenB in the biosynthesis of selenoneine

Wei Huang[1,2,3], Jun Song[1,2,3], Tianxue Sun[2], Yue He[2], Xiang Li[1], Zixin Deng[2], Feng Long[1,2]*

[1]Department of neurosurgery, Zhongnan Hospital of Wuhan University, School of Pharmaceutical Sciences, Wuhan University, Wuhan 430071, China

[2]Ministry of Education Key Laboratory of Combinatorial Biosynthesis and Drug Discovery, School of Pharmaceutical Sciences, Wuhan University, Wuhan 430071, China

[3]These authors contributed equally: Wei Huang, Jun Song

*Correspondence: longfe@whu.edu.cn

Table of Contents

Supplementary Table 1. Data collection and refinement statistics of SenB in complex with different sugar donors. 3

Supplementary Table 2. Detailed interactions between SenB and UDP. 4

Supplementary Table 3. DNA coding sequences and amino acid sequences of *Cb*SenB and *Rs*SenB. 5

Supplementary Table 4. Data collection and refinement statistics of *Rs*SenB. 7

Supplementary Table 5. Primers used in this work....8

Supplementary Table 6. GenBank numbers of the GT-B type glycosyltransferases used in this work. 10

Supplementary Fig. 1. Profiles of the size exclusion chromatography and SDS-PAGE analysis of SenB, *Cb*SenB, and *Rs*SenB. 11

Supplementary Fig. 2. LC-MS analysis of the mBBr derivatives of the SenB-catalyzed products using different UDP-sugar donors. 12

Supplementary Fig. 3. Dissociation constants (K_d values) of SenB for binding of different UDP-sugars. 13

Supplementary Fig. 4. The crystal structures of SenB complexed with various sugar donors determined in this work. 14

Supplementary Fig. 5. Structural basis for UDP binding of SenB. 15

Supplementary Fig. 6. SenB prefers the UDP-form sugar donor. 16

Supplementary Fig. 7. Structural basis for SeP binding of SenB.. 17

Supplementary Fig. 8. Sequence alignment of GT-B type glycosyltransferases.. 18

Supplementary Fig. 9. The spatial localization of the His-Asp in the structures of SenB and other GT-B type GTs. 19

Supplementary Fig. 10. Profiles of the size-exclusion chromatography and SDS-PAGE analysis of the H58A, H58D and H58Q SenB mutants. 20

Supplementary Fig. 11. Electron density comparison for the side chains of R155 and K158 in the three complex structures of SenB. 21

Supplementary Fig. 12. Residue conservation analysis over 200 SenB homologous sequences of SenB. 22

Supplementary Fig. 13. The SenB homologs are located within the putative SEN biosynthetic gene clusters. 23

Supplementary Fig. 14. HPLC-UV/DAD analysis of the mBBr derivatives of the reaction products catalyzed by *Cb*SenB, *Rs*SenB and their mutants using different sugar donors. 24

Supplementary Table 1. Data collection and refinement statistics of SenB in complex with different sugar donors.

	**SenB/UDP-GlcNAc**	**SenB/UDP-GalNAc**	**SenB/UDP-Glc/PO_4^{3-}**
**PDB Entry**	8JJT	8JJQ	8JJN
Space group	$P2_1$	$P2_1$	$P2_1$
a, *b*, *c* (Å)	108.22, 56.38, 108.45	108.11, 56.38, 108.13	107.83, 55.79, 108.08
α, β, γ (°)	90, 91.11, 90	90, 91.01, 90	90, 90.94, 90
Resolution (Å)	36.55-1.88 (1.95-1.88)	48.68- 1.64 (1.70-1.64)	47.99- 1.98 (2.05-1.98)
Unique reflections	106607 (10530)	157919 (14143)	89964 (8920)
Redundancy	6.6 (6.6)	6.2 (3.9)	6.6 (5.9)
Completeness (%)	99.75 (99.57)	98.72 (88.63)	99.91 (99.94)
I/σ(I)	15.60 (2.77)	13.44 (2.09)	13.76 (1.98)
R_{merge}	0.08 (0.71)	0.08 (0.50)	0.11 (0.92)
$CC_{1/2}$	0.998 (0.856)	0.997 (0.716)	0.998 (0.66)
R_{work}/R_{free}	0.201/0.227	0.189/0.207	0.195/0.221
**No. atoms**			
Protein	7223	7218	7273
Ligands	117	117	123
***B*-factor (Å²)**			
Protein	26.03	18.86	29.97
Ligands	20.67	14.19	32.56
Bond length (Å)	0.008	0.007	0.009
Bond angle (°)	1.05	1.02	0.96
Favored (%)	96.85	97.27	97.60
Allowed (%)	2.73	2.10	1.88
Outliers (%)	0.42	0.63	0.52

*Values in parentheses refer to the highest resolution shell.

Supplementary Table 2. Detailed interactions between SenB and UDP.

Residue	Residue atom	Ligand moiety	Ligand atom	Distance (Å)
**Hydrogen bond**				
R155	NH2	β-phosphate	O2B	2.7
K158	NZ		O2B	3.2
G19	N		O1B	3.2
H235	ND1/N	α-phosphate	O1A	2.8/2.8
V236	N		O2A	3.3
R22	NH1/NE	Ribose ring	O3C	3.0/4.0
V236	OE2		O3C	2.6
E239	OE1		O2C	2.9
N17	ND2		O2	2.8
L209	O/N	Uracil ring	N3/O4	3.2/3.5
T214	OG1		N3	3.3
**hydrophobic interaction**				
V151	CG1	Uracil ring	C5	3.7

Supplementary Table 3. DNA coding sequences and amino acid sequences of *Cb*SenB and *Rs*SenB.

***Cb*SenB (from *C. bacterium*, DNA coding sequences)**

atgggcagcagccatcatcatcatcatcacagcagcggcctggtgccgcgcggcagccatatgagccgtagcagtag
tgcagtgattgtgagtccggccctggccgatgcaaataatggtaattggcgcaccgcccagcgttggcagcagatgctg
gccccgcagcgccctgtgcgtattgttcgcgaatggccggatgcacaggcagccagcgatggtgtgatgctggcactg
catgcccgtcgcagtgcagcagcagtgcgtgcctggagtgaagcacatccgggccgtggcctggccgttgtgctgac
aggtaccgatctgtatcaggatctggcaaccagcgcagaagcacgtcatagtgtggccgtggcccagcgcctggttgt
gctgcaggaacgcggcgttgaagcactggatgcaccgctgcgtccgaaagcccgtgtgatttatcagagtggtccggc
ctggccgccgctggcaaaacctcgtgatggcctgggcgcagtgagtgttggtcatctgcgtgcagtgaaaaccccgca
gaccctgtttgaagcagcacgtctgatgcgcgatcgtgcagatattcgtattacccatattggtgatggcggtggtgaacc
gctgctggcacagcaggcacgtgatacccagcgcgattgcccgggttatgaatggctgggtgccctgccgcatgatgc
aaccctgcagcgcattcgcgaagcacatgtgctggttcataccagtgccatggaaggtggcgcccatgtgattctggaa
gcagttcgctgcggcaccccggtgctggctagtcgtgtggatggcaatgttggcatgctgggtgcagattatgaaggtta
ttttccgcatggcgatgcagcagcactggcacgcctgctgcaggattgccgtgcaacccagagtgataatccggcccc
gggtctgctggatcgcctggcagcacagtgtgcactgcgcgccccgctgtttgatgccgatgccgaacgtcgtgcact
gctgaatctgctgcaggaactggaaccgaccccgtaa

***Cb*SenB (from *C. bacterium*, amino acid sequences)**

MGSSHHHHHHSSGLVPRGSHMSRSSSAVIVSPALADANNGNWRTAQRWQQ
MLAPQRPVRIVREWPDAQAASDGVMLALHARRSAAAVRAWSEAHPGRGL
AVVLTGTDLYQDLATSAEARHSVAVAQRLVVLQERGVEALDAPLRPKARVI
YQSGPAWPPLAKPRDGLGAVSVGHLRAVKTPQTLFEAARLMRDRADIRITHI
GDGGGEPLLAQQARDTQRDCPGYEWLGALPHDATLQRIREAHVLVHTSAM
EGGAHVILEAVRCGTPVLASRVDGNVGMLGADYEGYFPHGDAAALARLLQ
DCRATQSDNPAPGLLDRLAAQCALRAPLFDADAERRALLNLLQELEPTP

***Rs*SenB (from *Ramlibacter sp.*, DNA coding sequences)**

atgggcagcagccatcatcatcatcatcacagcagcggcctggtgccgcgcggcagccatatgagtcgcccgagcgt
ggcaattgtgagtccggccgtggcaagcgccaataatggcaattggcagaccgcccgccgttggcaggaatttctgga
tggcacctgcaatgttcgtatgacccagcgctggccggatgatggcagtcaggatgatgttgttatgctggccctgcatg

cacgtcgcagtgcagatagcattgaagcatgggccagtgtgcatggcgatcgtggtctggcagttgttctgaccggtac

cgatctgtatcaggatattgtggtggatccgcgtgcccgccatagtctggaactggccggtcagctggttgttctgcagg

atctgggcgcagaagcactgccgccggcactgcgtggcaaaacccgtgtgatttatcagagcaccccgagccaggca

gcagccagcaaaccggataccgtgctgcaggcactgatggtgggccatctgcgtgaagttaaaagtccgcagaccct

gtttcaggcagcccgcctgctggccggtcatgatgatattcgcattgatcatattggtgaagccctggatccggttctggg

cgaacaggccctggcaacccagcgcgattgcccgaattatcgctggctgggtgcactgccgcatgatggtacccgcg

aacgcattcgttgcgcacatctgctggtgcatgccagcgcaatggaaggtggcgcccatgtgattatggaagccgtgtg

cagtggcaccccggttctggccagccgtattccgggtaatgtgggcatgctgggcgccgattatgcaggttattttaccc

atggcgatgcagccgccctggcagcactgctggttcgttgtcgtcagggtcaggccgccagtggtgatgttccggcag

atccgctgctggcacgcctgggtgcacagtgcgccctgcgtgcaccgctgtttgccccggaagcagaacgcgcagcc

ctgctgcgtctggtggccgatctgatgtaa

***Rs*SenB (from *Ramlibacter sp.*, amino acid sequences)**

MGSSHHHHHHSSGLVPRGSHMSRPSVAIVSPAVASANNGNWQTARRWQEFL

DGTCNVRMTQRWPDDGSQDDVVMLALHARRSADSIEAWASVHGDRGLAV

VLTGTDLYQDIVVDPRARHSLELAGQLVVLQDLGAEALPPALRGKTRVIYQ

STPSQAAASKPDTVLQALMVGHLREVKSPQTLFQAARLLAGHDDIRIDHIG

EALDPVLGEQALATQRDCPNYRWLGALPHDGTRERIRCAHLLVHASAMEG

GAHVIMEAVCSGTPVLASRIPGNVGMLGADYAGYFTHGDAAALAALLVRC

RQGQAASGDVPADPLLARLGAQCALRAP LFAPEAERAALLRLVADLM*

Supplementary Table 4. Data collection and refinement statistics of *Rs*SenB.

	***Rs*SenB**
**PDB Entry**	8K5U
Space group	$P2_1$
a, *b*, *c* (Å)	80.83, 73.79, 81.28
α, β, γ (°)	90, 116.11, 90
Resolution (Å)	35.39-2.15 (2.23-2.15)
Unique reflections	46812 (4638)
Redundancy	6.6 (5.9)
Completeness (%)	99.84 (99.98)
I/σ(I)	10.21 (2.47)
R_{merge}	0.15 (0.69)
$CC_{1/2}$	0.994 (0.813)
R_{work}/R_{free}	0.206/0.250
**No. atoms**	
Protein	4808
Ligands	0
***B*-factor (Å^2)**	
Protein	24.90
Ligands	0
Bond length (Å)	0.008
Bond angle (°)	0.91
Favored (%)	96.68
Allowed (%)	3.01
Outliers (%)	0.32

*Values in parentheses refer to the highest resolution shell.

Supplementary Table 5. Primers used in this work.

Primer name	Sequence (5′-3′)
N17A-F	GCGCTGCCGGGCGCGGCAAACGGCAACTGGCGC
N17A-R	GCGCCAGTTGCCGTTTGCCGCGCCCGGCAGCGC
G19A-F	CCGGGCGCGAACAACGCAAACTGGCGCACCGCT
G19A-R	AGCGGTGCGCCAGTTTGCGTTGTTCGCGCCCGG
N20A-F	GGCGCGAACAACGGCGCATGGCGCACCGCTCAA
N20A-R	TTGAGCGGTGCGCCATGCGCCGTTGTTCGCGCC
R22A-F	AACAACGGCAACTGGGCAACCGCTCAACGCTGG
R22A-R	CCAGCGTTGAGCGGTTGCCCAGTTGCCGTTGTT
T23A-F	AACGGCAACTGGCGCGCAGCTCAACGCTGGAAA
T23A-R	TTTCCAGCGTTGAGCTGCGCGCCAGTTGCCGTT
H58A-F	GTGATGCTGGCGCTGGCAGCGCGCCGCAGCGCG
H58A-R	CGCGCTGCGGCGCGCTGCCAGCGCCAGCATCAC
H58Q-F	GTGATGCTGGCGCTGCAGGCGCGCCGCAGCGCG
H58Q-R	CGCGCTGCGGCGCGCCTGCAGCGCCAGCATCAC
H58D-F	GTGATGCTGGCGCTGGATGCGCGCCGCAGCGCG
H58D-R	CGCGCTGCGGCGCGCATCCAGCGCCAGCATCAC
R61A-F	GCGCTGCATGCGCGCGCAAGCGCGGAAAGCATT
R61A-R	AATGCTTTCCGCGCTTGCGCGCGCATGCAGCGC
T83A-F	TGGGCGTGGTGCTGGCAGGCACCGATCTGTATC
T83A-R	ATACAGATCGGTGCCTGCCAGCACCACGCCCAG
T85A-F	GTGGTGCTGACCGGCGCAGATCTGTATCAAGAT
T85A-R	ATCTTGATACAGATCTGCGCCGGTCAGCACCAC
Q131A-F	GCCCGCGTGGTGTATGCAAGCACGAGCGCGCGT
Q131A-R	ACGCGCGCTCGTGCTTGCATACACCACGCGGGC
V151A-F	AGCTGCGCGCGGTGATGGCAGGCCATCTGCGCC
V151A-R	AGATGGCCTGCCATCACCGCGCGCAGCTGACG
H153A-F	TGATGGTGGGCGCACTGCGCCAAGTGAAAAGCCCGC

H153A-R	TTCACTTGGCGCAGTGCGCCCACCATCACCGCGCGC
R155A-F	ATGGTGGGCCATCTGGCACAAGTGAAAAGCCCG
R155A-R	CGGGCTTTTCACTTGTGCCAGATGGCCCACCAT
K158A-F	CATCTGCGCCAAGTGGCAAGCCCGCAGACCCTG
K158A-R	CAGGGTCTGCGGGCTTGCCACTTGGCGCAGATG
K158R-F	CATCTGCGCCAAGTGCGTAGCCCGCAGACCCTG
K158R-R	CAGGGTCTGCGGGCTACGCACTTGGCGCAGATG
K158H-F	CATCTGCGCCAAGTGCATAGCCCGCAGACCCTG
K158H-R	CAGGGTCTGCGGGCTATGCACTTGGCGCAGATG
K158E-F	CATCTGCGCCAAGTGGAAAGCCCGCAGACCCTG
K158E-R	CAGGGTCTGCGGGCTTTCCACTTGGCGCAGATG
K158N-F	CATCTGCGCCAAGTGAATAGCCCGCAGACCCTG
K158N-R	CAGGGTCTGCGGGCTATTCACTTGGCGCAGATG
K158G-F	CATCTGCGCCAAGTGGGTAGCCCGCAGACCCTG
K158G-R	CAGGGTCTGCGGGCTACCCACTTGGCGCAGATG
K158V-F	CATCTGCGCCAAGTGGTTAGCCCGCAGACCCTG
K158V-R	CAGGGTCTGCGGGCTAACCACTTGGCGCAGATG
I181A-F	AAGATATTCGCATTGATCATGCAGGGGACGCG
I181A-R	CGCGTCCCCTGCATGATCAATGCGAATATCTTCGC
L209A-F	ATGGTTGGGCGCGGCACCGCATGCGCAGACCCG
L209A-R	TGCGCATGCGGTGCCGCGCCCAACCATCTGTACCC
T214A-F	CTGCCGCATGCGCAGGCACGTCAGCGCATTCAG
T214A-R	CTGAATGCGCTGACGTGCCTGCGCATGCGGCAG
E231A-F	CATACGAGCGCGCTGGCAGGCGGCGCGCATGTG
E231A-R	CACATGCGCGCCGCCTGCCAGCGCGCTCGTATG
H235A-F	CTGGAAGGCGGCGCGGCAGTGATTATGGAAGCG
H235A-R	CGCTTCCATAATCACTGCCGCGCCGCCTTCCAG
E239A-F	GCGCATGTGATTATGGCAGCGGTGCGCAGCGGC
E239A-R	GCCGCTGCGCACCGCTGCCATAATCACATGCGC

Supplementary Table 6. GenBank numbers of the GT-B type glycosyltransferases used in this work.

Protein	GenBank number
UGT74AN1	MF942416.1
UGT74AN2	MF942417.1
UGT74AN3	MF942418.1
*Gg*CGT	QGL05036.1
*Zm*CGTa	NP_001132650.2
*Lp*CGTa	MK894451.1
*Lp*CGTb	MK894452.1
*Sb*CGTa	MK894443.1
*Sb*CGTb	MK894444.1
UGT708C1	AB909375.1
UGT74AC1	K7NBW3.1
UGT74AC2	MF417499.1
*Pa*GT2	AB368371.1
*Pa*GT3	AB368372.1
*Tc*CGT1	MK644229.1
UGT76G1	KM206772.1
UGT89C1	NP_563756.1
*Pt*UGT1	LC475438.1
UGT74F2	MT416756.1
*Os*79	XP_015635481.1
UGT78K6	BAF49298.1
UGT85H2	XP_003618665.1
Ct3GTa	AB185904
UGT72B1	XM_037324960.1
*Vv*GT1	NM_001397857.1
UGT71G1	AY747627.1
UGT78G1	XP_003610163.1

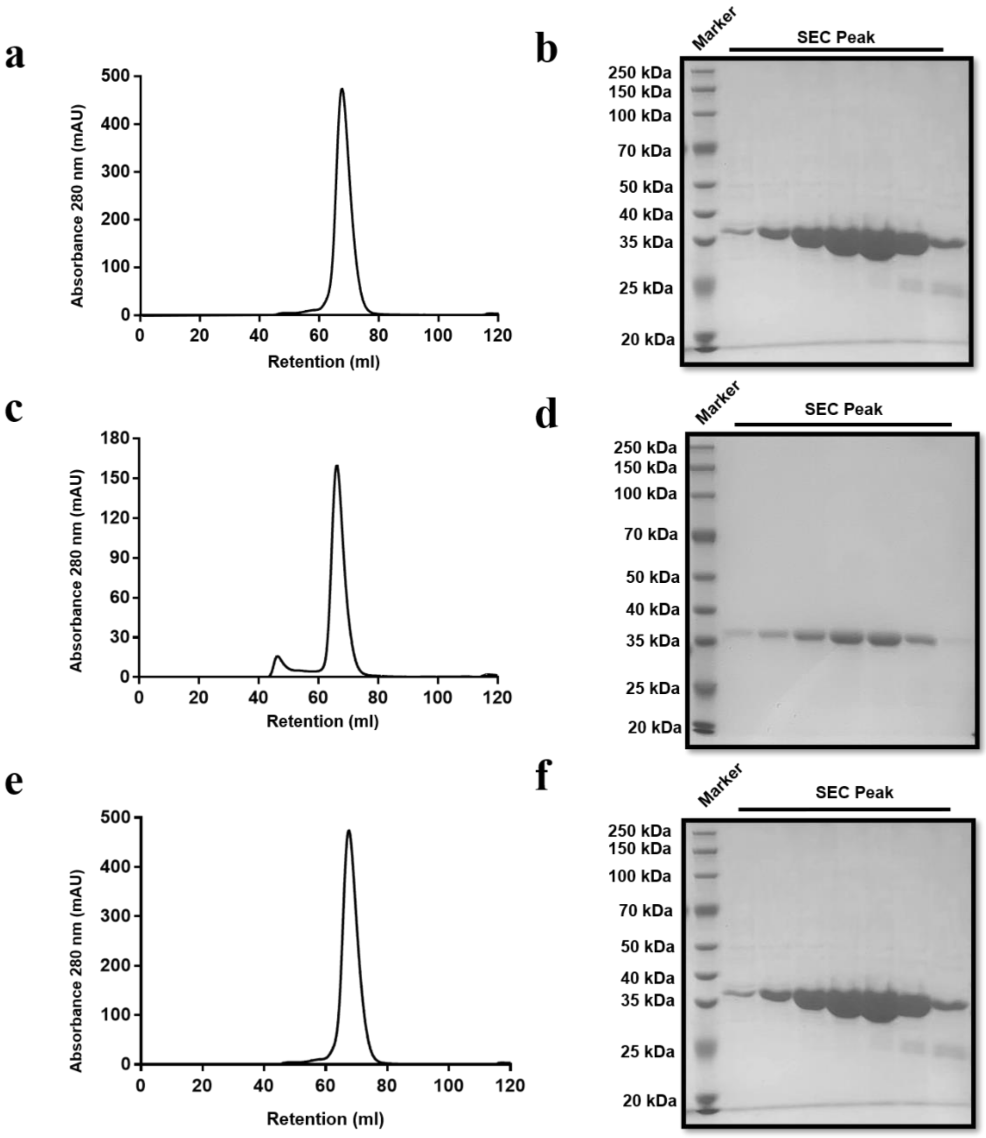

Supplementary Fig. 1. Profiles of the size exclusion chromatography and SDS-PAGE analysis of SenB, *Cb*SenB, and *Rs*SenB. a, Size-exclusion chromatography of SenB. **b,** SDS-PAGE analysis of the purified SenB. **c,** Size-exclusion chromatography of *Cb*SenB. **d,** SDS-PAGE analysis of the purified *Cb*SenB. **e,** Size-exclusion chromatography of *Rs*SenB. **f,** SDS-PAGE analysis of the purified *Rs*SenB. The source data for 1a, 1c and 1e are provided in a Source Data file. The uncropped scans of the gels in 1b, 1d and 1f are supplied at the end of the Supplementary Information file.

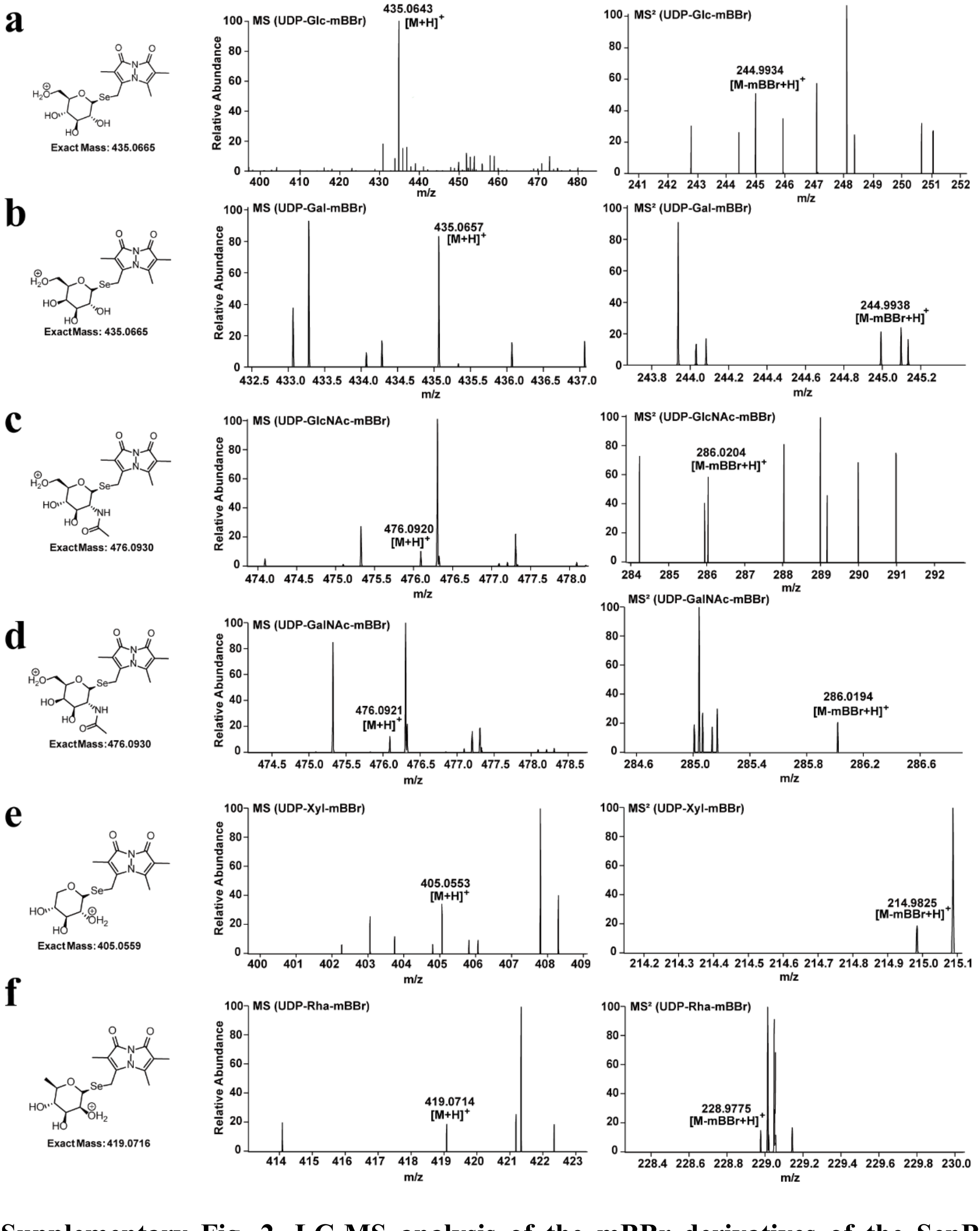

Supplementary Fig. 2. LC-MS analysis of the mBBr derivatives of the SenB-catalyzed products using different UDP-sugar donors. a, (+) ESI MS and MS/MS spectra for UDP-Glc-mBBr. **b,** (+) ESI MS and MS/MS spectra for UDP-Gal-mBBr. **c,** (+) ESI MS and MS/MS spectra for UDP-GlcNAc-mBBr. **d,** (+) ESI MS and MS/MS spectra for UDP-GalNAc-mBBr. **e,** (+) ESI MS and MS/MS spectra for UDP-Xyl-mBBr. **f,** (+) ESI MS and MS/MS spectra for UDP-Rha-mBBr.

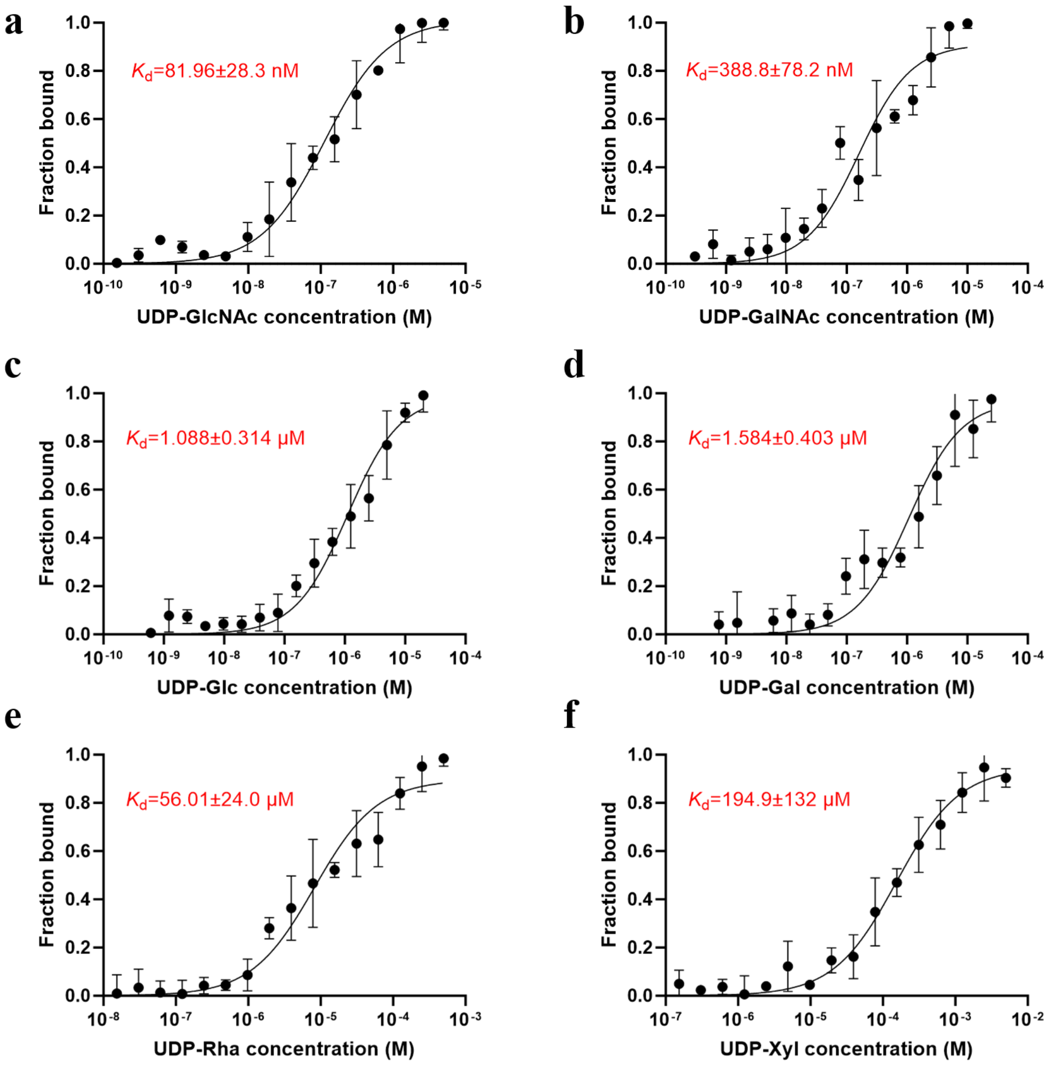

Supplementary Fig. 3. Dissociation constants (*K*d values) of SenB for binding of different UDP-sugars. a, UDP-GlcNAc. **b,** UDP-GalNAc. **c,** UDP-Glc. **d,** UDP-Gal. **e,** UDP-Rha. **f,** UDP-Xyl. Data are presented as mean values ± SD (n = 3 independent experiments). Source data are provided as a Source Data file.

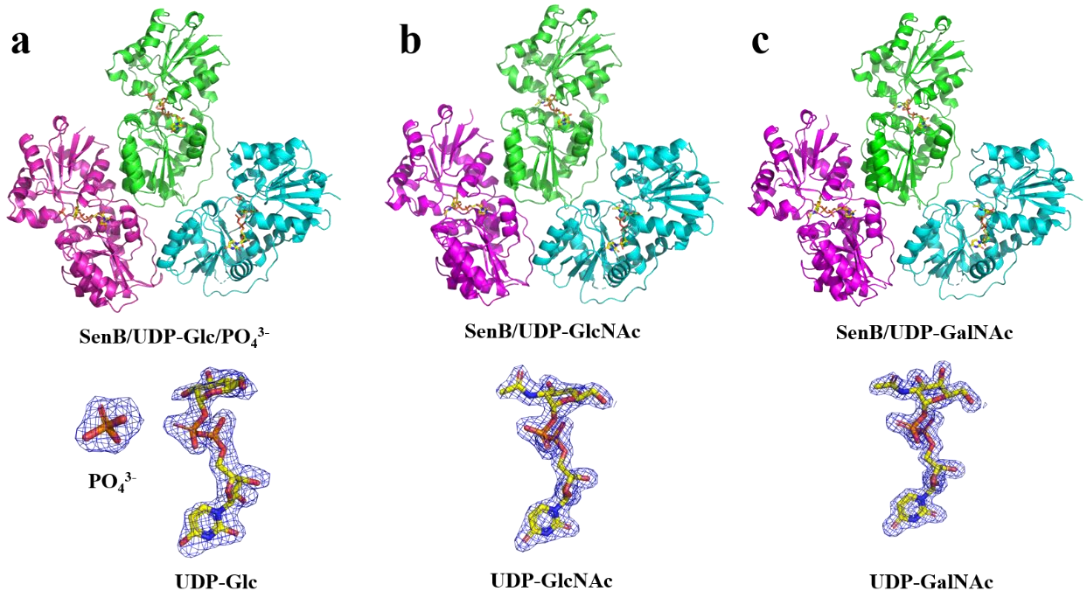

Supplementary Fig. 4. The crystal structures of SenB complexed with various sugar donors determined in this work. Cartoon diagrams of the structures of SenB/UDP-Glc/PO_4^{3-} (**a**), SenB/UDP-GlcNAc (**b**), and SenB/UDP-GalNAc (**c**) are shown in the upper panel. In the bottom panel, the corresponding bound ligands, including UDP-Glc and PO_4^{3-} (**a**), UDP-GlcNAc (**b**), and GalNAc (**c**), are shown in the F_o-F_c omit electron density maps contoured at 2.0 σ, respectively.

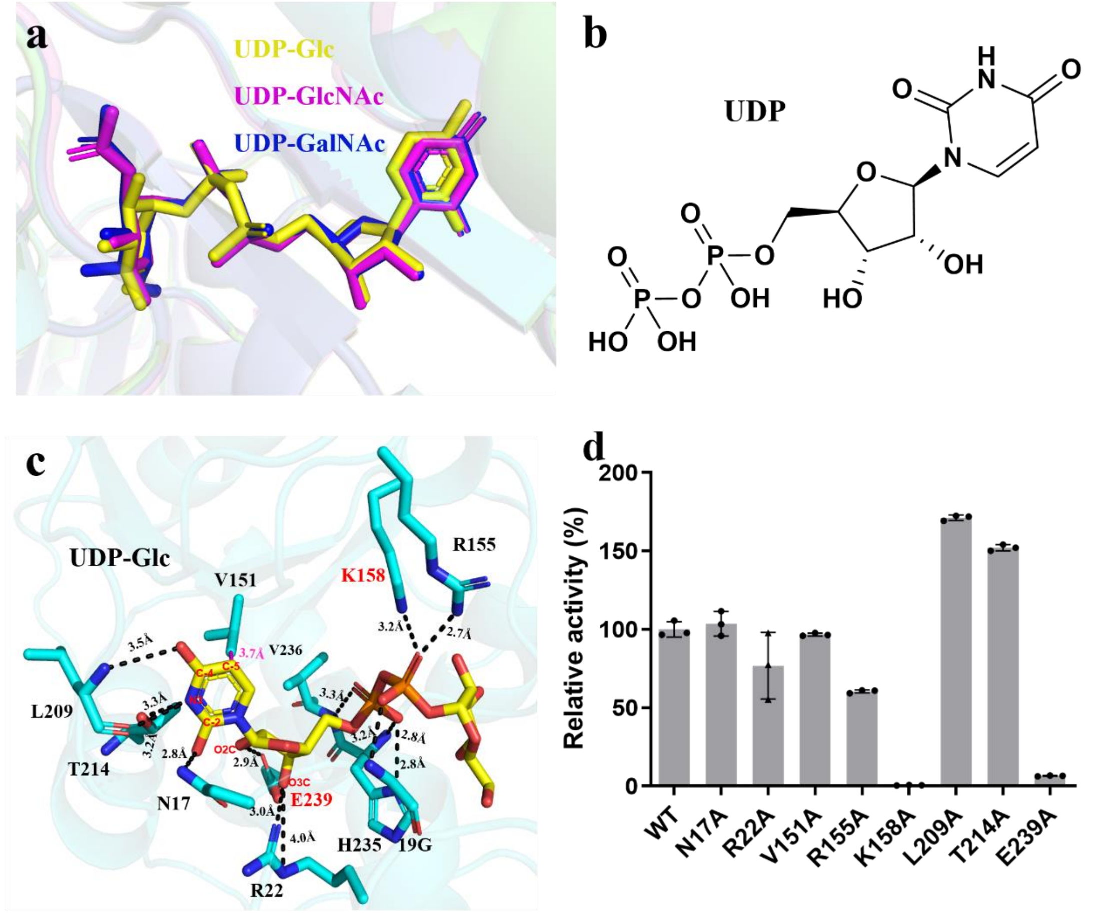

Supplementary Fig. 5. Structural basis for UDP binding of SenB. a, Structural superposition of three SenB complex structures. **b,** Chemical structure of UDP. **c,** Interaction between UDP and SenB. **d,** Relative catalytic activities of affected by the mutations potentially involved in the SenB-UDP interaction. Data are presented as mean values ± SD (n = 3 independent experiments). Source data are provided as a Source Data file.

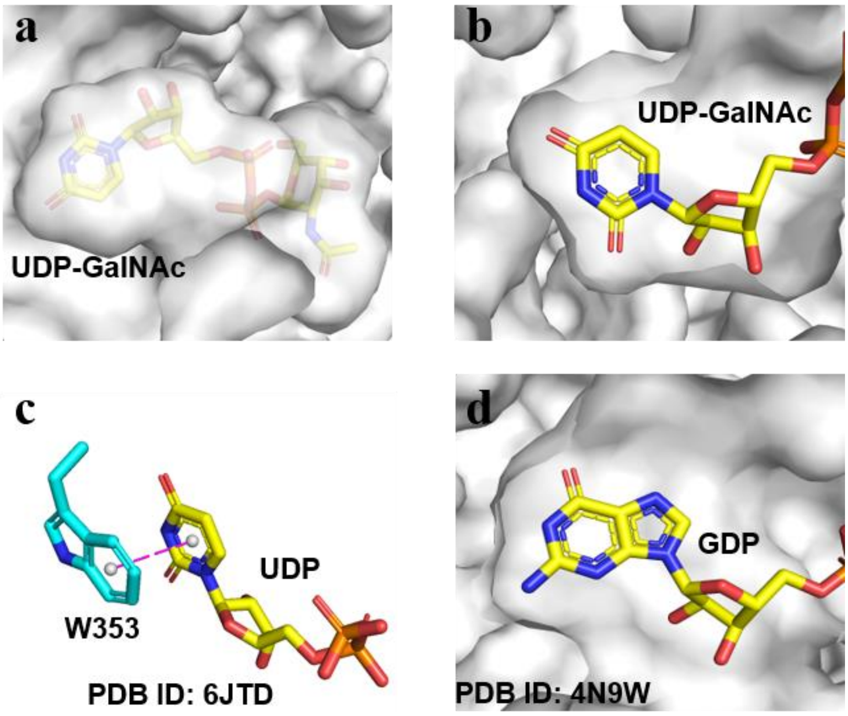

Supplementary Fig. 6. SenB prefers the UDP-form sugar donor. a, Surface view of the bound UDP-GalNAc in SenB. **b,** Sectional view of the bound UDP-GalNAc in SenB. **c,** π-π interaction between UDP and *Tc*CGT1 (PDB ID: 6JTD), a representative of the GT-B type GT. **d,** Sectional view of the bound GDP in PimA (PDB ID: 4N9W), a GT-B type GT that can recognize the GDP-form sugar donor.

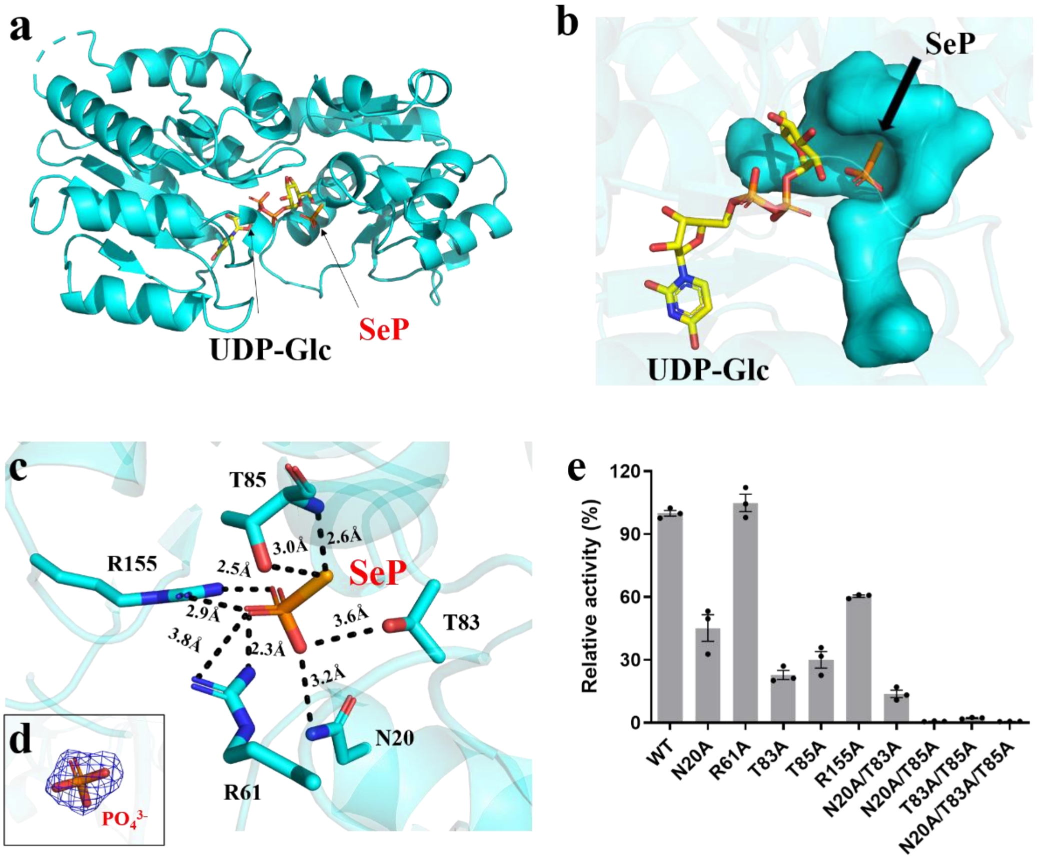

Supplementary Fig. 7. Structural basis for SeP binding of SenB. a, The ternary complex structure of SenB/UDP-Glc/SeP. **b,** The narrow SeP binding pocket of SenB. **c,** Interaction between SeP and SenB. **d,** F_o-F_c omit electron density map of PO_4^{3-} contoured at 2.0σ. **e,** Relative catalytic activity of the SenB mutants using UDP-Glc as the sugar donor. Data are presented as mean values ± SD (n = 3 independent experiments). Source data are provided as a Source Data file.

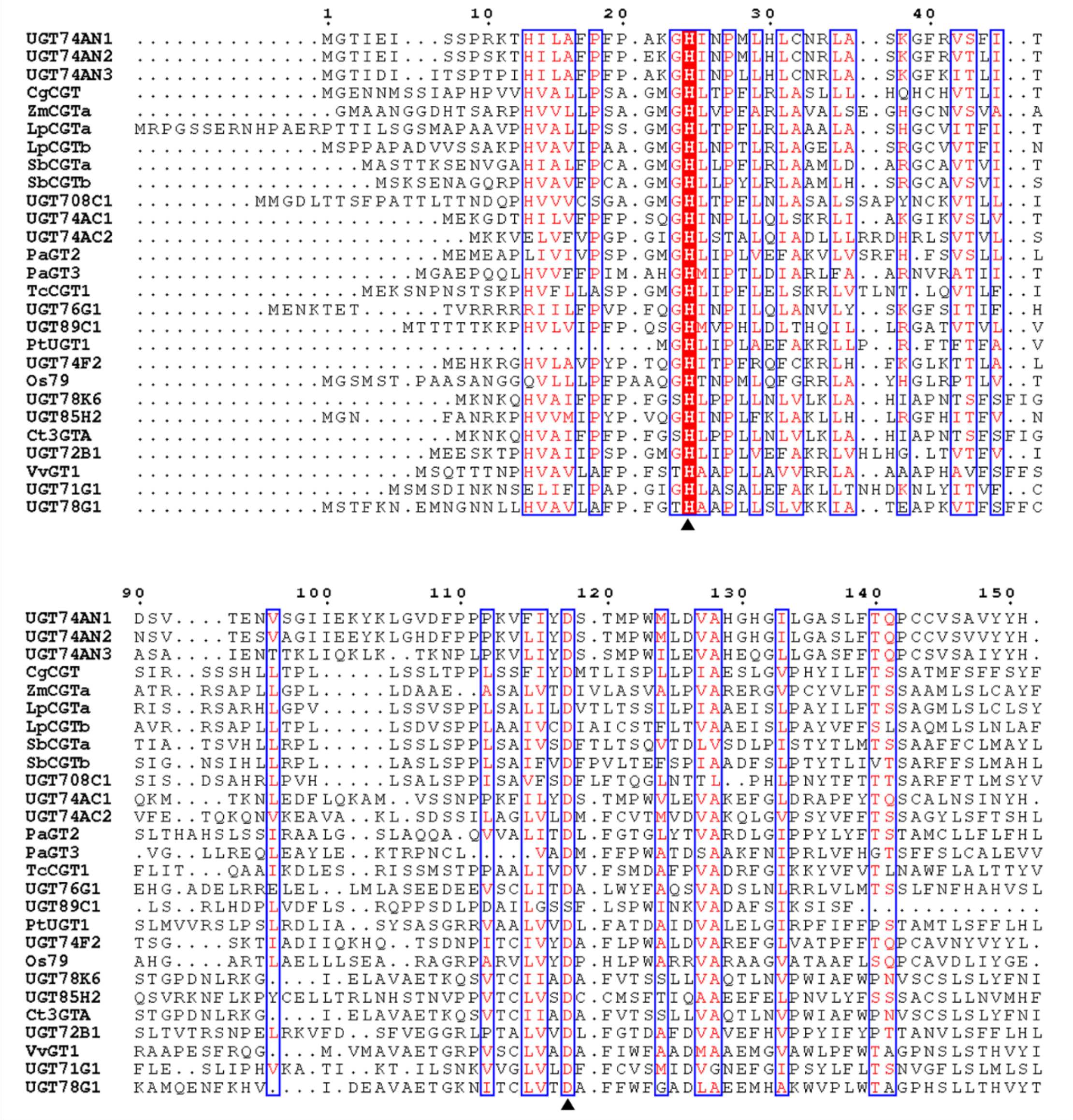

Supplementary Fig. 8. Sequence alignment of GT-B type glycosyltransferases. The catalytic dyad His-Asp was labeled with black triangles. The GenBank numbers of these glycosyltransferase were listed in the Supplementary Table 6.

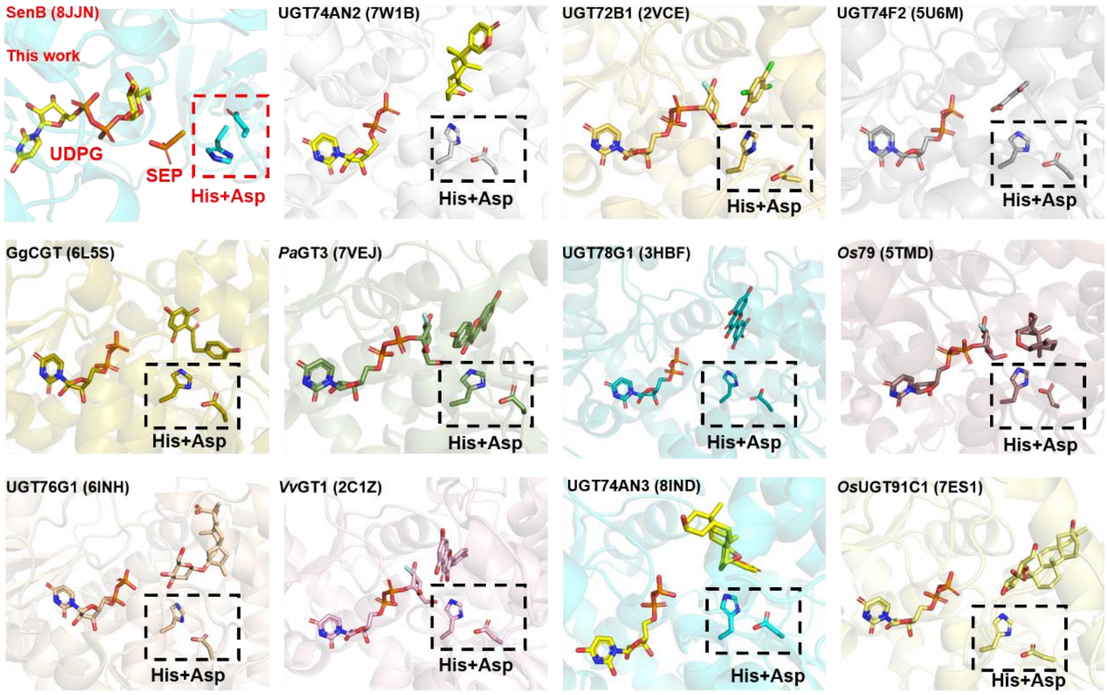

Supplementary Fig. 9. The spatial localization of the His-Asp in the structures of SenB and other GT-B type GTs.

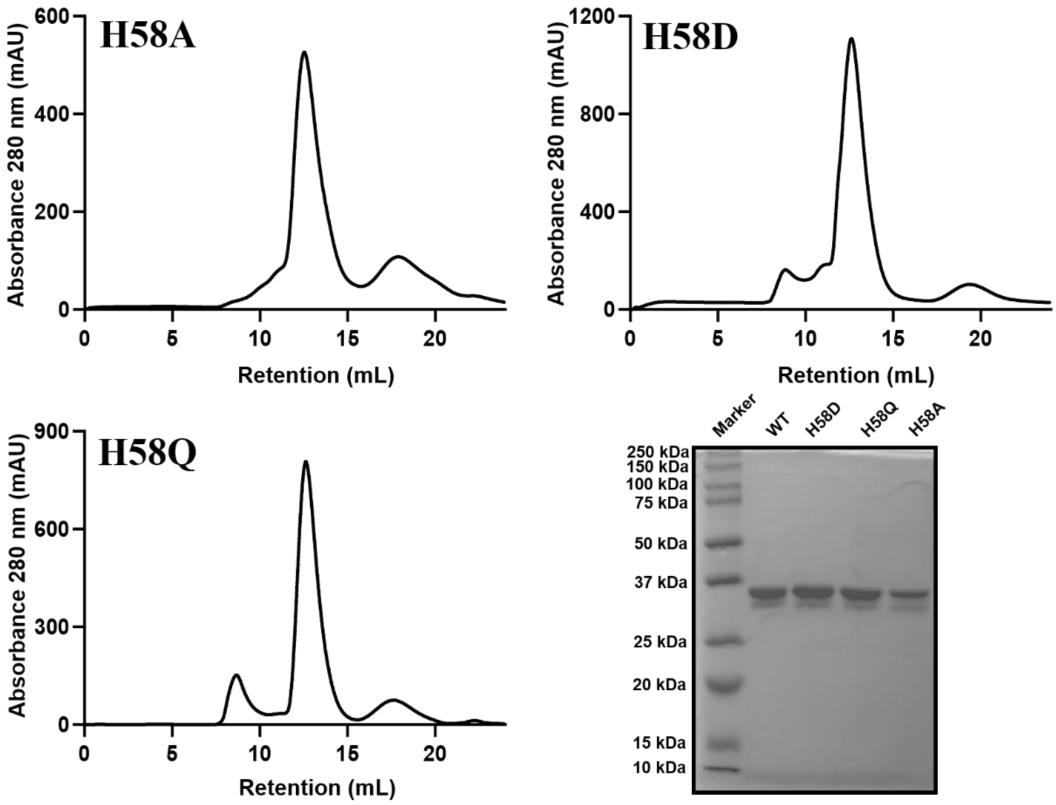

Supplementary Fig. 10. Profiles of the size-exclusion chromatography and SDS-PAGE analysis of the H58A, H58D and H58Q SenB mutants. Source data are provided as a Source Data file. The uncropped scan of the gel is supplied at the end of the Supplementary Information file.

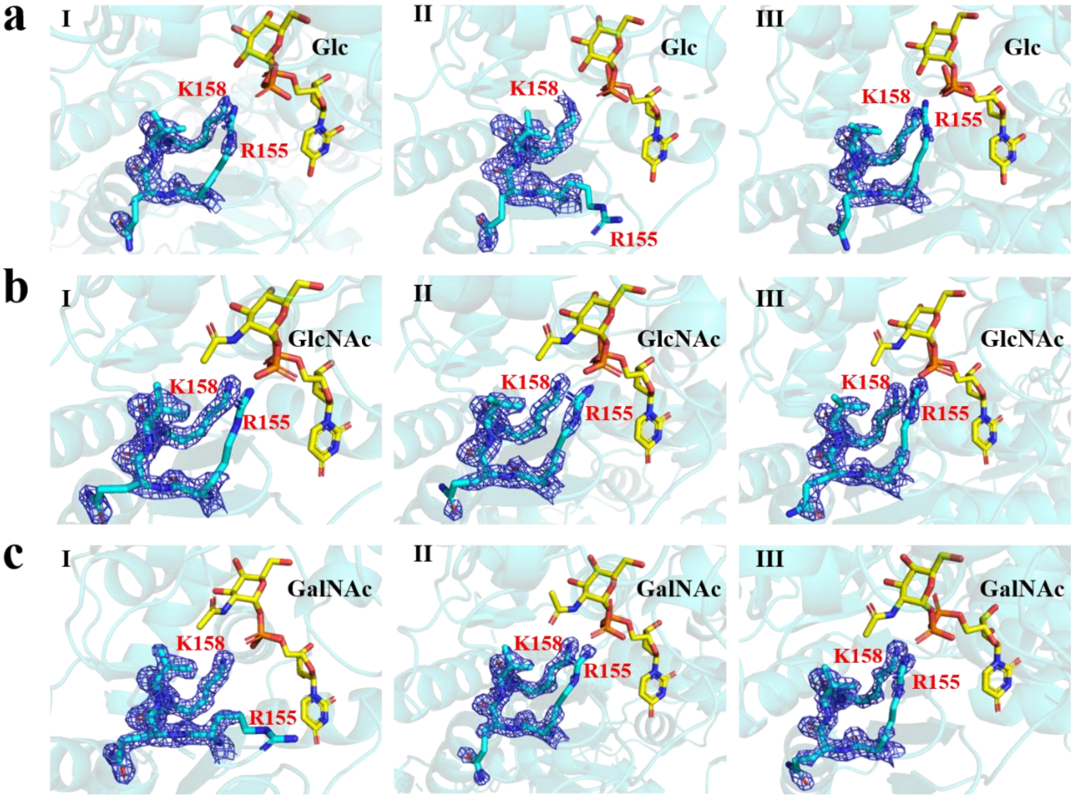

Supplementary Fig. 11. Electron density comparison for the side chains of R155 and K158 in the three complex structures of SenB. The electron density maps ($2F_o$-F_c, contoured at 2.0σ) of R155 and K158 of the three SenB monomers (I, II, III) from the complexes of SenB/UDP-Glc/PO_4^{3-} (**a**), SenB/UDP-GlcNAc (**b**), and SenB/UDP-GalNAc (**c**).

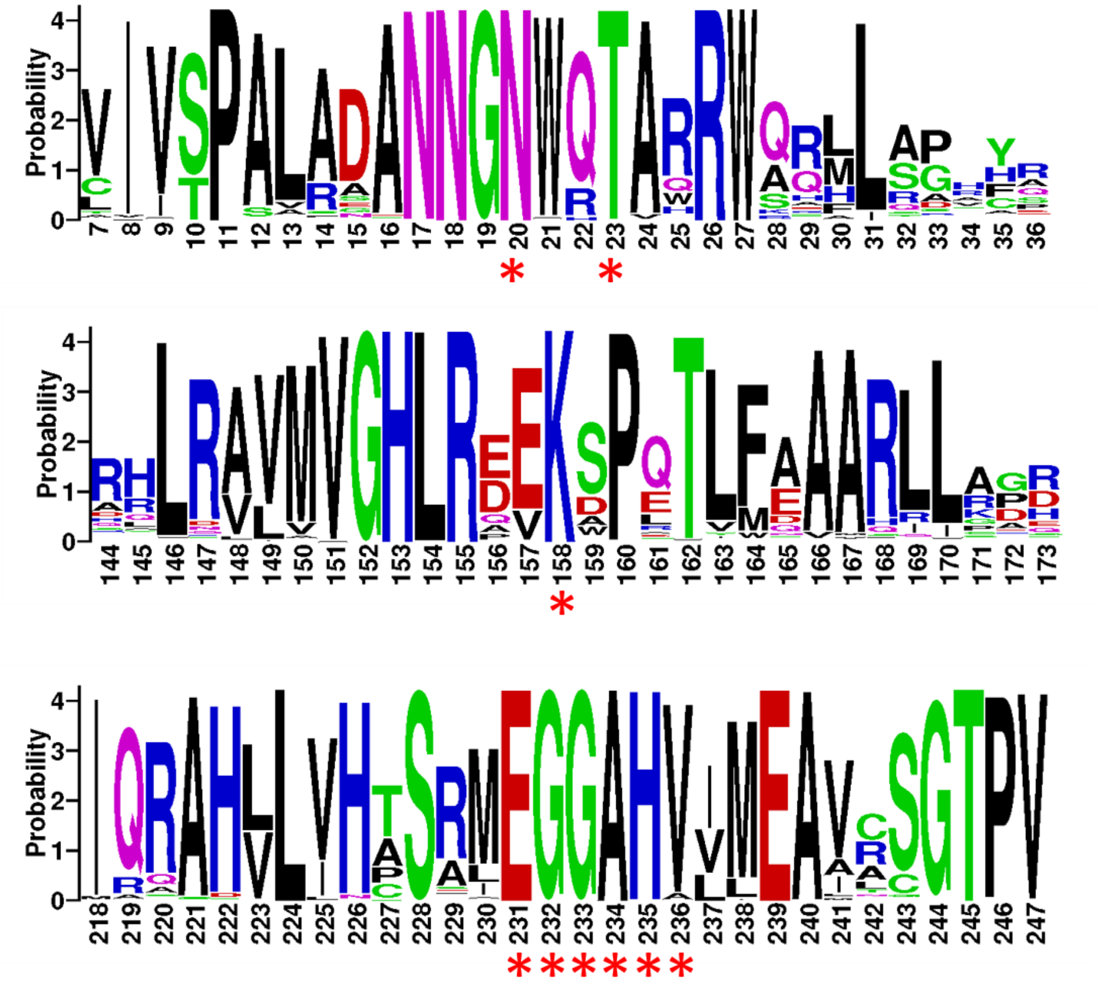

Supplementary Fig. 12. Residue conservation analysis over 200 SenB homologous sequences of SenB. The conserved residues N20/T23, H58/D86/K158, and EGGAHV are labeled with red asterisks.

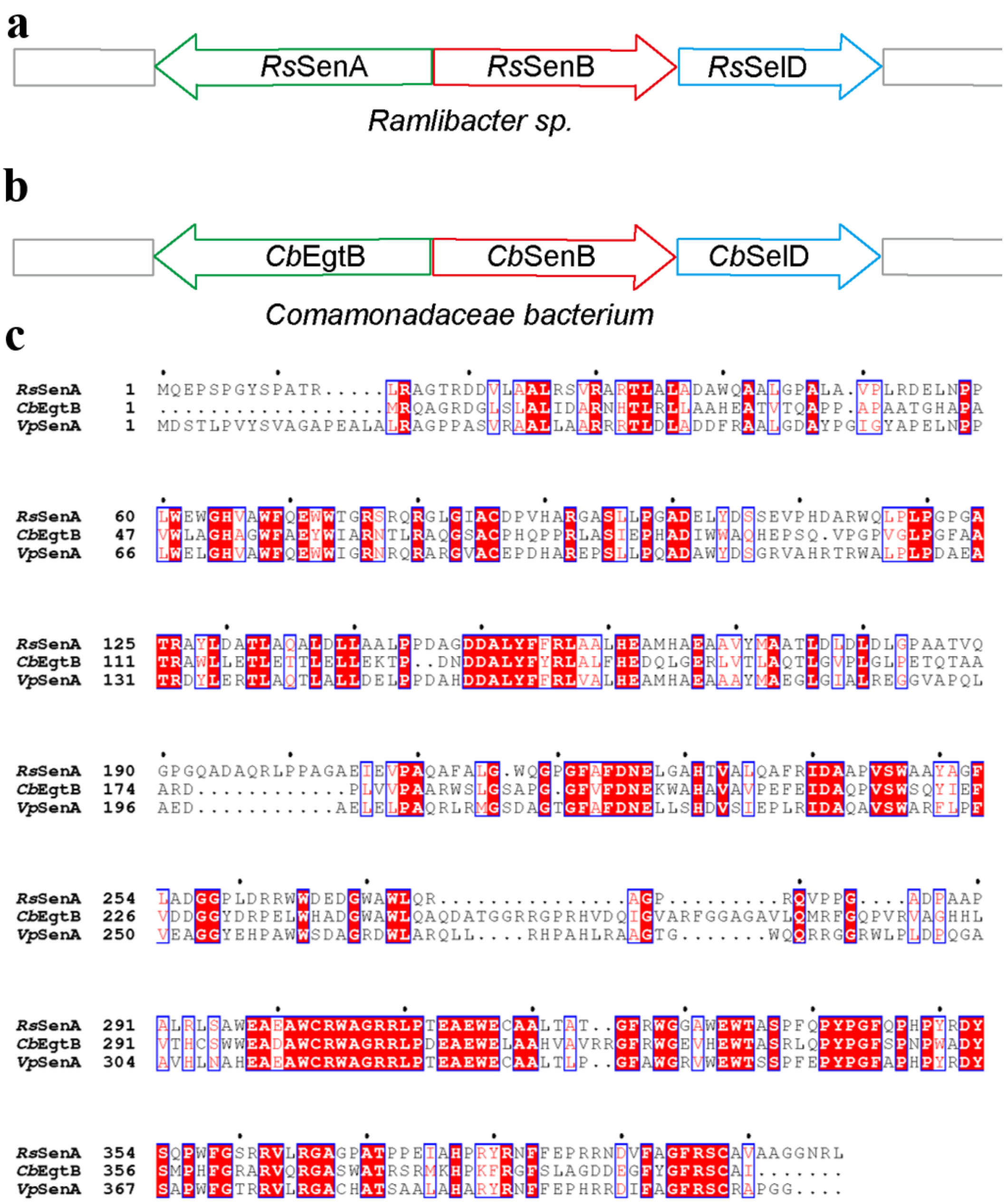

Supplementary Fig. 13. The SenB homologs are located within the putative SEN biosynthetic gene clusters. a, the putative SEN biosynthetic gene cluster containing *Rs*SenB in *Ramlibacter sp*, **b**, the putative SEN biosynthetic gene cluster containing *Cb*SenB in *Comamonadaceae bacterium* **c,** Protein sequence alignment of *Rs*SenA, *Cb*EgtB and *Vp*SenA.

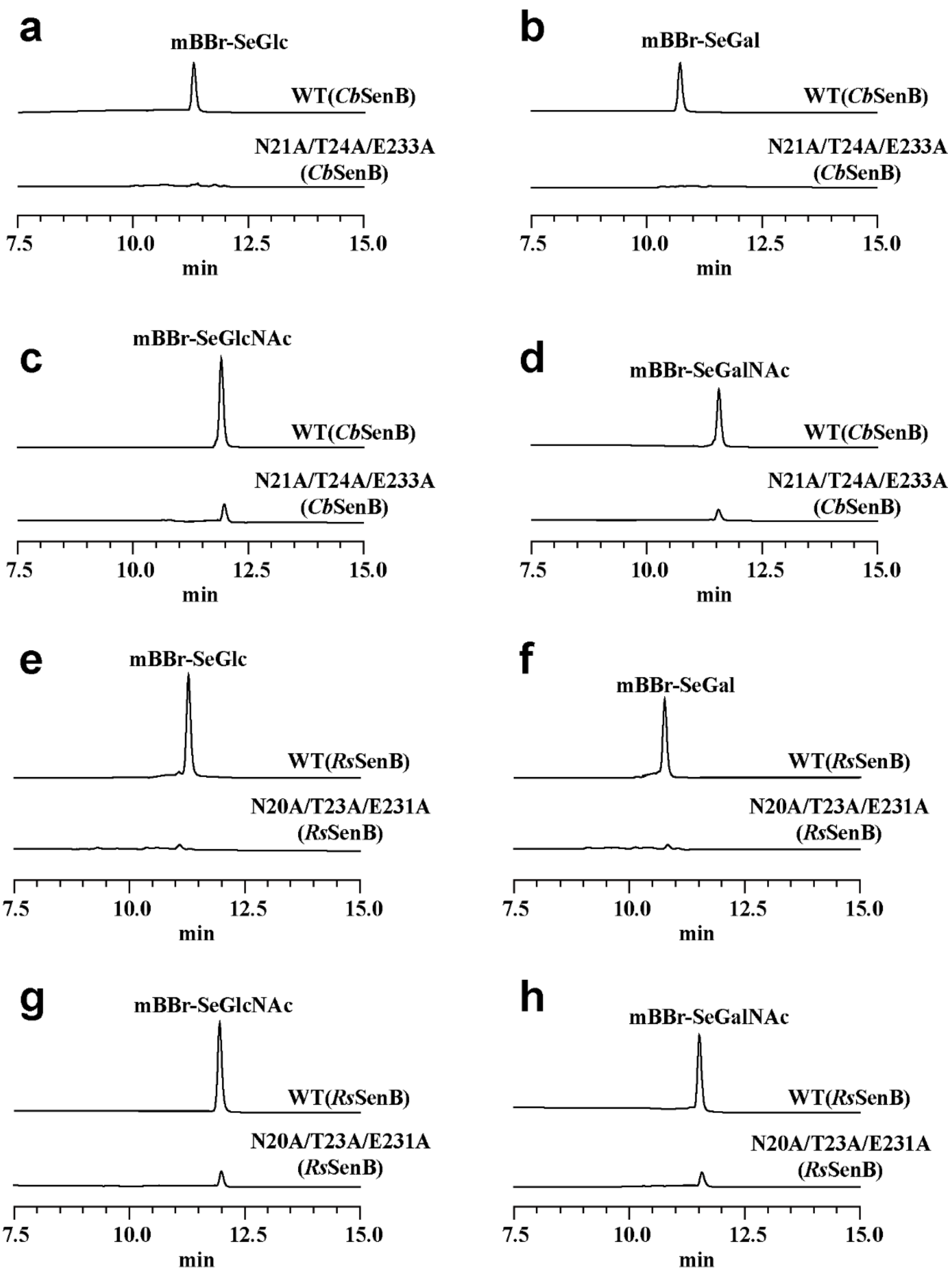

Supplementary Fig. 14. HPLC-UV/DAD analysis of the mBBr derivatives of the reaction products catalyzed by *Cb*SenB, *Rs*SenB and their mutants using different sugar donors. a-d, Product detection of *Cb*SenB and its triple mutant N21A/T24A/E233A using UDP-Glc (**a**), UDP-Gal (**b**), UDP-GlcNAc (**c**), or UDP-GalNAc (**d**) as the sugar donor. **e-f,** Product detection of *Rs*SenB and its triple mutant N20A/T23A/E231A using UDP-Glc (**e**), UDP-Gal (**f**), UDP-GlcNAc (**g**), or UDP-GalNAc (**h**) as the sugar donor.

Supplementary Figure 1b

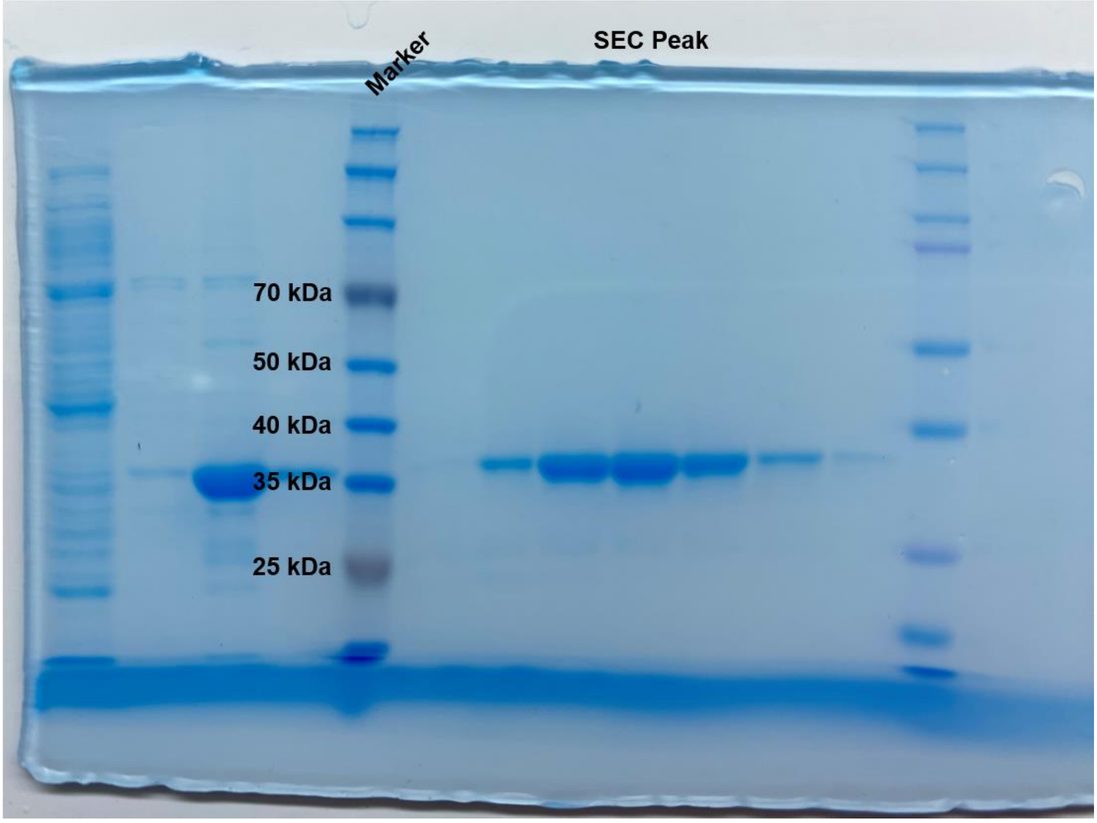

Supplementary Figure 1d

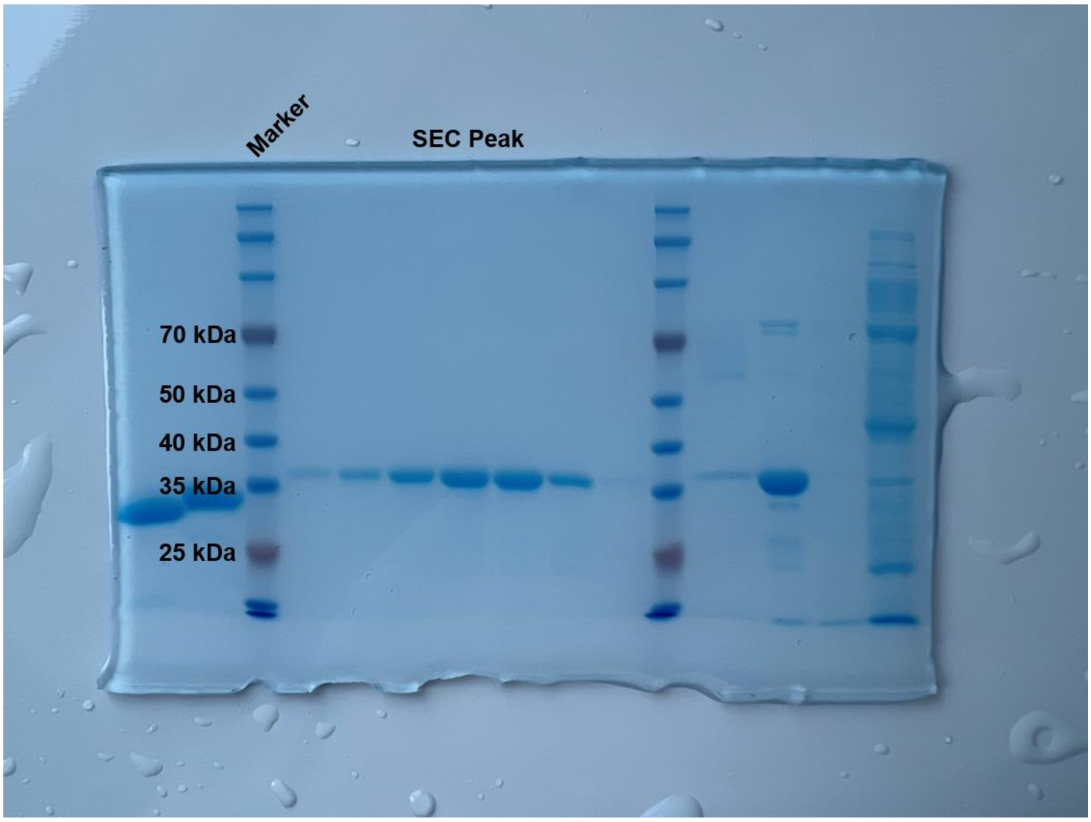

Supplementary Figure 1f

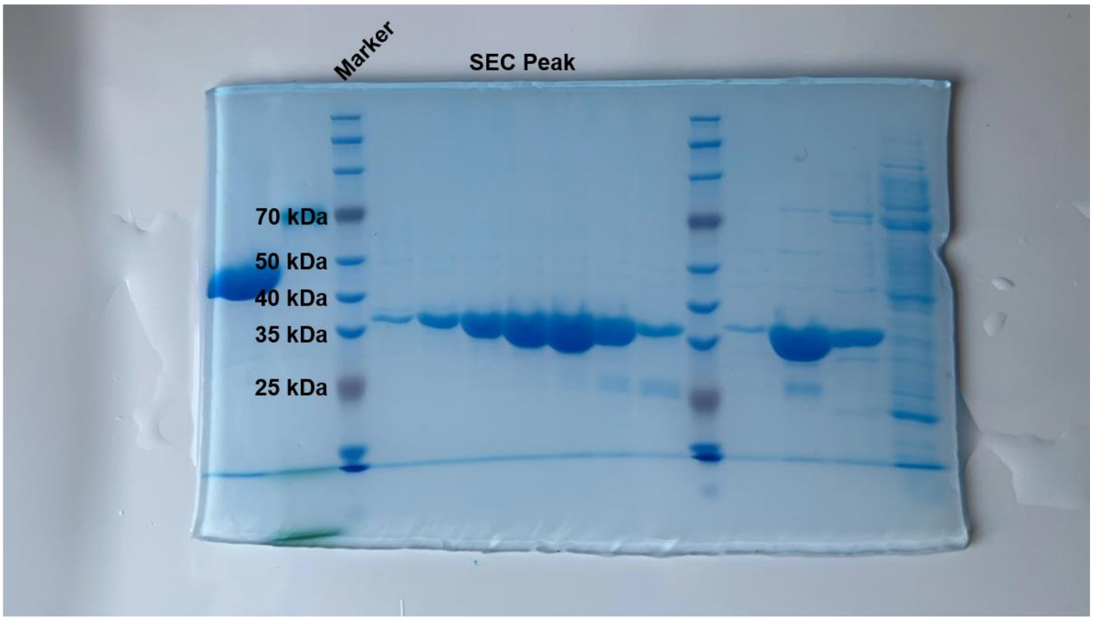

Supplementary Fig. 10

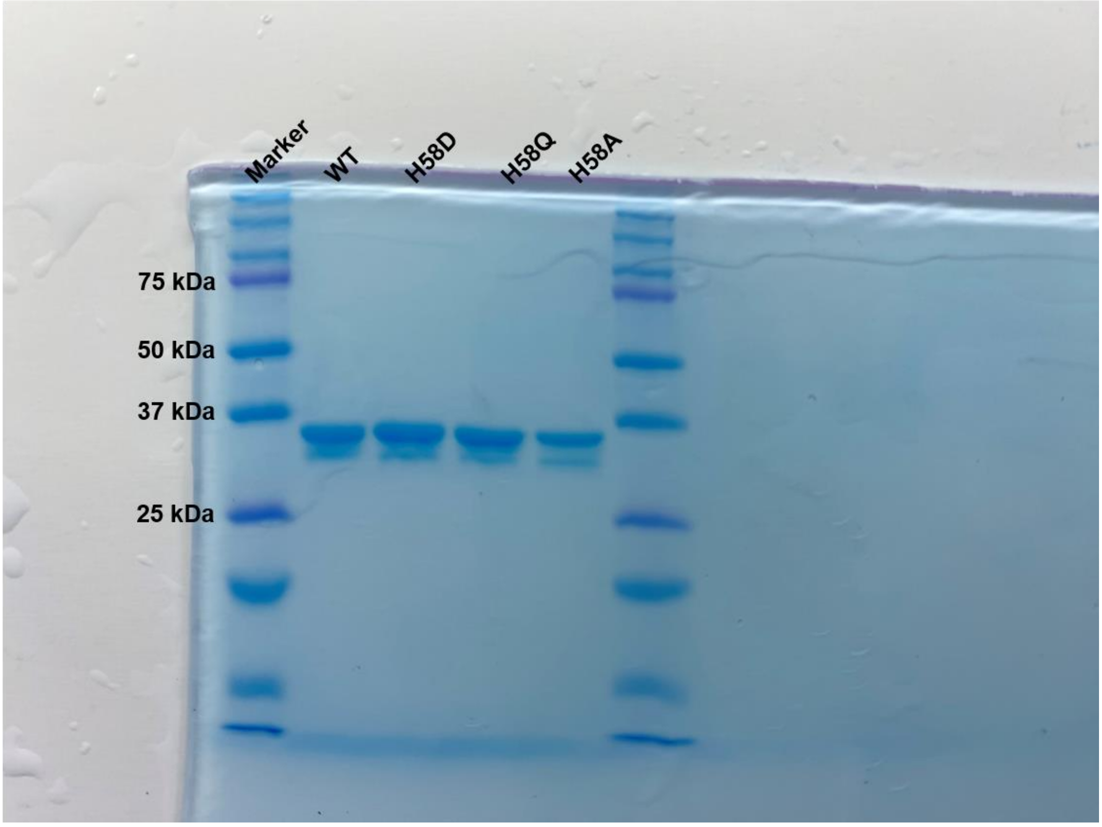

Supplement: Supplementary file 1 — Supplementary Information [file 41467_2024_46065_MOESM1_ESM.pdf]
